# Concealing the Taste of Fish-Oil

**Published:** 1849-10

**Authors:** 


*Concealing the taste of Fish-Oil.*—Now that the swallowing of cod-liver oil bids fair to become the fashionable mania of the day, it may be well to state the simple and effectual means communicated by M. Fredericq, of disguising its abominable taste. This merely consists in masticating a morsel of dried orange-peel just before and just after swallowing the dose.—*Rev. Med.-Chir.*, t. v., p. 114.

